# Getting Blood out of a Stone: Vascularization via Spheroids and Organoids in 3D Bioprinting

**DOI:** 10.3390/cells14090665

**Published:** 2025-05-01

**Authors:** Daria Revokatova, Polina Bikmulina, Zahra Heydari, Anna Solovieva, Massoud Vosough, Anastasia Shpichka, Peter Timashev

**Affiliations:** 1 Institute for Regenerative Medicine, I. M. Sechenov First Moscow State Medical University, 119991 Moscow, Russia; 2 Semenov Institute of Chemical Physics, 119991 Moscow, Russia; 3 Regenerative Medicine Department, Royan Institute for Stem Cell Science, Tehran 16635148, Iran

**Keywords:** organoid, spheroid, vascularization, 3D bioprinting

## Abstract

Current developments in bioequivalent technology have led to the creation of excellent models that mimic the structure and function of human organs. These models are based on the original tissues and organs of the human body, but they lack the complex interaction with the extensive network of vasculature, and this is a major challenge for these models. A functional vasculature is essential for oxygen, nutrient, and waste exchange. It is also responsible for inductive biochemical exchange, and provides a structural pattern for organ growth. In vitro systems, containing no perfusable vessels, suffer from the quick formation of a necrotic core of organoids, and further development does not occur due to increased metabolic demands. Another key limitation of 3D-based techniques is the absence of accurate architectural structures and large-scale tissue sizes. Recently, new 3D bioprinting methods have been developed for organoids and spheroids as living building blocks. These methods aim to address some of the challenges associated with 3D technologies. In this review, we discuss recent strategies for vascularization via organoids and spheroids, which are used as structural units in bioprinting to recreate natural organs and tissues with ever-increasing accuracy in structure and function.

## 1. Introduction

Tissue engineering is an actively developing field providing new solutions for therapy and enabling the creation of adequate models for testing the effectiveness and safety of personalized medicine. Bioprinting technology is a key area within tissue engineering, and represents one of the most promising approaches for forming bioequivalents. It allows for the creation of complex three-dimensional structures composed of different cell types embedded in the specific extracellular matrix (ECM). Currently, bioprinting is employed to develop a wide range of models and bioequivalents for various organs. However, despite the advantages of this method, challenges remain, particularly regarding the lack of vascularization in the formed bioequivalents. All tissues need to be supplied with nutrients and oxygen and cleaned out from waste products through a highly branched vasculature network. The distance between a living cell and the capillary supplying it must not exceed the oxygen diffusion limit (100–200 μm). While rather small tissue equivalents fall within this range and usually integrate well in the host vasculature, larger constructs often face severe cell death due to the lack of oxygen and nutrients [1]. Therefore, the creation of functional vascular networks is crucial for regenerative medicine, especially for the long-term maintenance of engineered 3D tissue constructs.

In vivo vascularization occurs through angiogenesis or vasculogenesis. Vasculogenesis happens during the embryo development when mesodermal cells differentiate into angioblasts, and then into endothelial cells. In angiogenesis, new vessels sprout from a pre-existing vessel [2] in response to pro-angiogenic growth factors [3]. When it comes to vessel formation in vitro, we mostly stimulate angiogenesis. However, vasculogenesis can also occur when cells are co-cultured with mesodermal cells.

Bottom-up tissue engineering approaches have been indicated as a new opportunity to assemble living building blocks into modified tissue architectures [4,5]. Using 3D-assembled structures, including organoids, spheroids or cell-laden microgels, for bioprinting can be a promising tool for the formation of a vascularized tissue [6], and they have been widely studied in various fields including cancer research, disease modeling, and drug development. Unlike a cell suspension, organoids and spheroids can recapitulate more realistic physiological conditions, such as the following in vivo microenvironments: cell–cell and cell–extracellular matrix (ECM) interactions, oxygen, and nutrient distribution (in limited sizes). Spheroids are widely used as a structural unit for bioprinting, since they are viable, size-controllable, and have numerous ways to produce a high number of standard structures. In some cases, using organoids can be better, because the signaling pathways leading to the organoid formation are like those present during the in vivo organ development [7,8,9,10]. However, the use of organoids for 3D bioprinting is currently limited due to the lack of a standardized and simple method for their creation.

For the successful assembly of organoids and spheroids through bioprinting, many parameters including the exogenous chemical environment, cell-specific self-organization, bioprinting method, biomaterials, and efficient vascularization of the fabricated tissues should be considered [11,12].

The aim of this review is to develop a practical strategy for the creation of a vascularized tissue using spheroids and organoids by bioprinting.

## 2. Spheroids and Organoids

The process of achieving an effective vascular network and anastomosis—the connection between the blood vessels of the host and those of the tissue-engineered graft—is particularly complex when relying solely on materials. However, incorporating living cells into the design of these artificial constructs can greatly facilitate this process, since the formation of anastomoses is driven from two distinct points. Numerous studies have demonstrated the advantages of using cell aggregates over suspensions of individual cells in various aspects, including viability, preservation of phenotype, and biomimicry. Cell aggregates tend to exhibit improved survival rates and functionality, as they better mimic the natural architecture and behavior of tissues. This enhanced performance is crucial for ensuring that artificial organs or tissues can effectively integrate with the patient’s existing vascular system. Because of their simplicity, spheroids are the most commonly used type of cell aggregates.

Spheroids represent cell aggregates, [13], and are now becoming more commonly used as a bioink component [14] since they can be produced with a uniform diameter and a narrow size distribution [15,16]. Furthermore, they represent a universal model for a wide variety of organs and tissues, such as the cartilage, pancreas, cardiac muscle, pituitary gland, optic cup, and the layered cortical tissue [17]. Also, spheroids can be used as a center for vascularization, and have proved their effectiveness in different tissue models [18]. For instance, cells from the dissociated postnatal rodent cortex showed spontaneous organization of endothelial cells in capillaries with lumens [19]. The cell position in a spheroid can also influence the resulting sprouting pattern and vasculogenic capacity. A core–shell spheroid with human umbilical vein endothelial cells (HUVECs) on the periphery and human turbinate mesenchymal stem cells (hTMSCs) inside exhibited longer sprouts, increased branching points, and CD31+ cells in comparison with spatially mixed structures [20]. Therefore, a proper spheroid’s spatial structure may allow the control of cell differentiation, while a 3D spheroid culture provides the ability to scale up the tissue construct and alleviate the graft’s integration with the host tissues. To make this possible, the key factors of a pre-vascularized spheroid’s formation, such as the cell composition, spatial organization, spheroid size, and other external and internal factors, should be considered.

The next step in the development of “building blocks” for bioprinting is organoids. They have a more complex cell composition and a pre-formed inner structure. Generally, organoids are defined as platforms initiating from pluripotent stem cells (ESCs or iPSCs), adult stem cells, progenitors, and tumor cells. These structures have a potential to self-organize and self-assemble into an in vivo-like organ with the corresponding physiology, and display the ability to repeat the organ’s functions. The terms “organoid” and “spheroid” are often used interchangeably, but “spheroid” is a more general term, which describes an aggregate of cells, while organoids are more complex and can recapitulate the structure, cellular behavior, and functions of more complex organs. Organoids are also used as a structural unit for complex tissue bioprinting [21,22]. Despite their promising features, organoids present some limitations, including a limited level of maturity and function, heterogeneity, technically complicated culturing, monitoring, etc.

Due to their limitations in nutrient and oxygen diffusion, these platforms only support growth on a millimeter-scale [23]. By integrating the vasculature, the size of organoids and spheroids could be significantly increased, and the vasculature’s endothelium can provide paracrine signaling to increase maturation [24]. To integrate complex cellular structures with the forming capillaries and vessels, several approaches can be applied.

## 3. Vascularization Strategies

**Figure 1:**
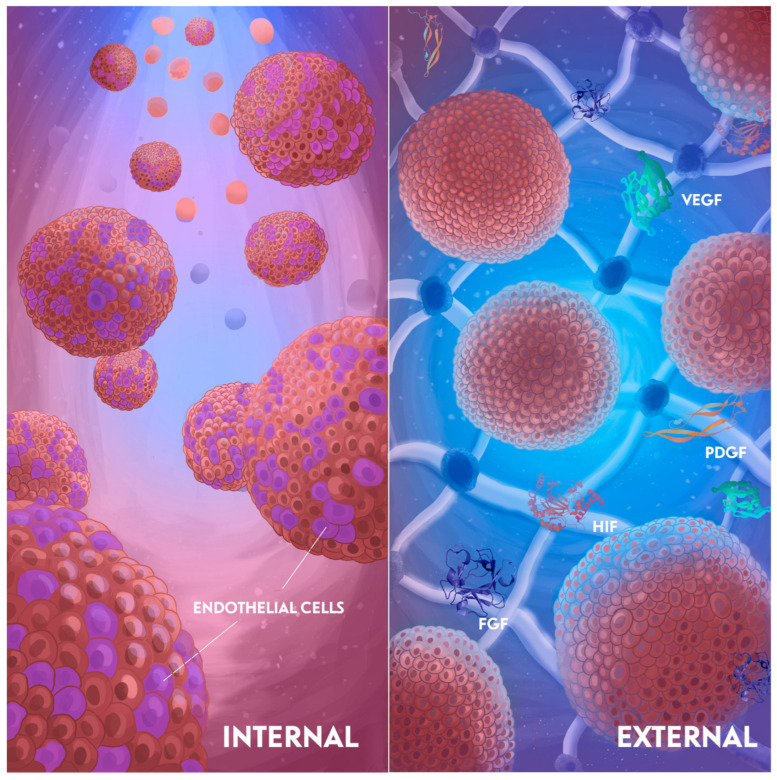
Vascularization strategies. Vascularization induction approaches can be divided in two groups: internal and external. Internal induction relies on cell differentiation and focuses on the careful selection of cell types for co-differentiation, primarily utilizing endothelial cells, as illustrated in the left image. In contrast, external induction focuses on external factors, such as the composition of the medium, growth factors, and the properties of hydrogels, and is depicted in the right image. Internal induction is driven by cell differentiation and focused on the accurate choosing of cell types for co-differentiation. Mostly, endothelial cells are used, and this is demonstrated in the (**left**) picture. External induction is focused on the medium composition, such as growth factors and hydrogel characteristics. This is demonstrated in the (**right**) picture. Often, these methods are used together to achieve the best vascularization results. PDGF—platelet-derived growth factor, VEGF—vascular endothelial growth factor, FGF—fibroblast growth factors, HIF—hypoxia-inducible factor.

Generally, approaches to vascularization induction can be divided in two groups: internal and external. These two strategies are connected, but originate from different sources. The internal induction is driven by inner cell processes, first of all differentiation (mediated by the cell types in a spheroid or organoid). This approach is focused on accurately choosing the cell types for co-differentiation, and then allowing them to self-organize. The external approach is aimed at vascularization through external factors—medium composition, substrate mechanical properties, and in vivo transplantation. Often, these methods are used together to achieve the best vascularization results. Nevertheless, the understanding of the separate factors driving vascularization is essential for the development of more functional tissue equivalents. The concept for fabricating pre-vascularized spheroids via the internal or external approaches is discussed below and illustrated in Figure 1.

### 3.1. Internal Induction

The design of a tissue-engineered construct starts by defining the cellular composition. The most popular way to provide a favorable environment for vascularization is the co-culturing of cells, predominantly endothelial cells. The general approach to form co-culture spheroids is the self-assembly of a cell mixture in non-adhesive wells, rotating vessels, or hydrogels [25,26]. In addition to promoting the formation of a vascularized construct, the co-culture with endothelial cells or their precursors is known to play an important role in the paracrine regulation, which can trigger cell differentiation. This is essentially important for bone tissue formation [27] and tumor modeling [28].

**Figure 2:**
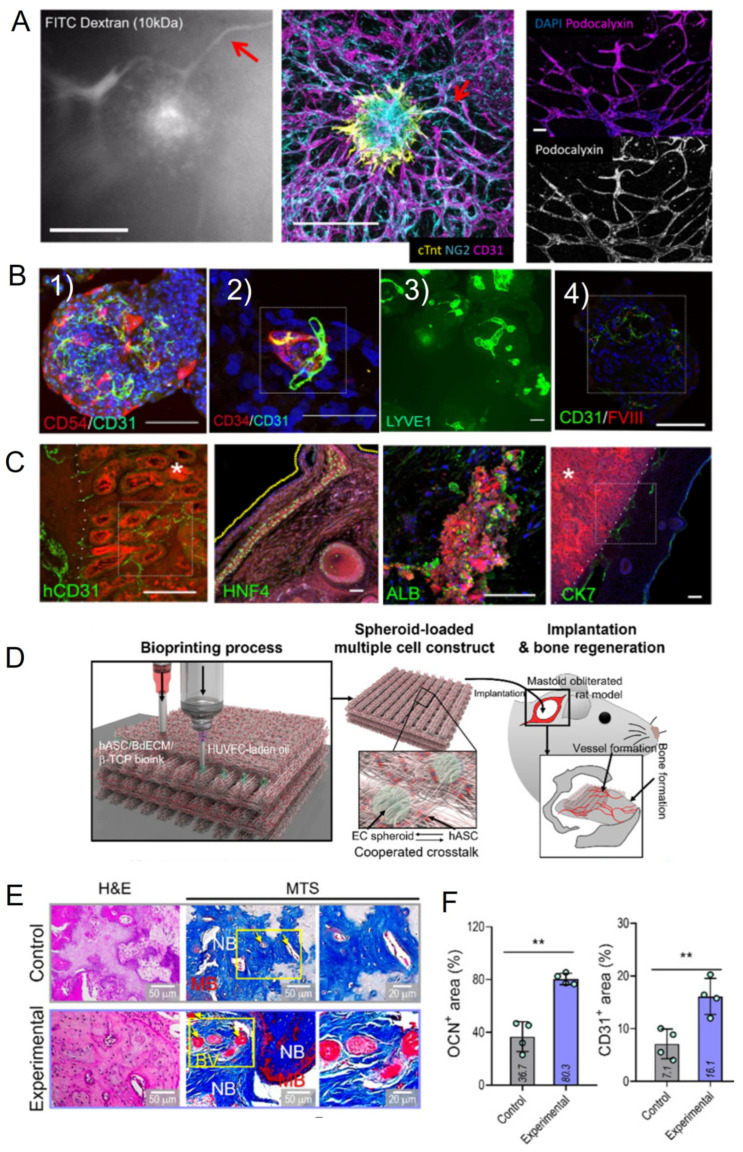
Examples of vascularized organoids application. (**A**) Cardiac spheroids from cardiomyocytes, endothelial cells (HUVECs), and fibroblasts cultured on microfluidic chip (µFCs) with perivascular cells—pericytes. Synthesis of cTnT (cardiac troponin T), NG2 (pericyte marker) and CD31 (ECs marker) (middle picture). Cardiac spheroids in µFCs are perfusable, which was investigated by FITC dextran assay (left) and positive podocalyxin staining (right) to prove the lumen formation. The red arrows indicate a structure that is proposed to correspond in the Dextran perfusion assay and the immunostaining image. The images were adapted and changed from Ref. [29] according to http://creativecommons.org/licenses/by/4.0/ (assessed on 27 April 2025). (**B**) Liver organoids from human primary hepatocytes, ESC, and iPSC showing expression of the endothelial markers CD54 and CD31 (1) and overlapping expression of CD34 and CD31 in a small structure indicate neovascularization. Scale bar, 50 μm (2). LYVE1, a sinusoidal endothelial marker, shows the diversity of lumen shapes and sizes (3). Co-localization of liver endothelial-associated FVIII and endothelial cell marker CD31 and luminal spaces in the organoids. Scale bar, 100 μm (4). (**C**) Immunostaining of mouse kidney/organoid transplant cryosections demonstrates the presence of human hepatic populations (CK7, hCD31, HNF4α, and ALB) in vitro. The boundary between the kidney parenchyma and organoid marked with *. A yellow dotted line marks the external surface. The images were adapted and changed from Ref. [30] according to http://creativecommons.org/licenses/by/4.0/ (assessed on 27 April 2025). (**D**) Application of ECs spheroids for formation of vascularized bone tissue. Schematic diagram of the fabrication process of EC spheroid and in vivo evaluation. (**E**) Histological staining images (hematoxylin and eosin (H&E) and Masson’s trichrome staining (MTS) of the implanted constructs at 8 weeks. Yellow arrows indicate developed vessels. New bone (NB), blood vessel (BV), and matured bone (MB). (**F**) OCN-positive and CD31-positive areas measured from the OCN and CD31 images. ** *p* < 0.01. The images were adapted and changed from Ref. [31] according to http://creativecommons.org/licenses/by/4.0/ (assessed on 27 April 2025).

The most popular approaches to the stimulation of internal angiogenesis using a co-culture system are described below, and some examples of vascularized spheroids and organoids are presented in Figure 2:

Co-culture with endothelial cells. The common approach to form spheroids and organoids suitable for the utilization as prevascularized blocks for various tissue engineering applications is co-culture with endothelial cells. This approach enables the reciprocal paracrine regulation and spatial cell organization [32].

There are some sources of endothelial cells (ECs) which are applied more often in the co-culture, including human umbilical vein ECs (HUVECs), endothelial colony–forming cell ECs (ECFC-ECs), endothelial progenitor cells (EPCs) from adult blood, hPSC-derived ECs, and some organ-specific ECs, such as liver sinusoidal endothelial cells (LSECs) [33,34]. Among them, HUVECs are extensively used in these efforts since they are easy-to-harvest from the fetal umbilical cord. Cocultivation with HUVECs showed excellent results when creating liver [35], bone tissue [36], cardiac tissue [29], and other constructs. Usually, HUVECs form a connected microvasculature inside spheroids, and can grow on flat surfaces or in hydrogels [20]. This perforating network allows an increase in the spheroid size up to 650 μm [37]. By varying the ratio of cells contained in spheroids, different goals can be achieved. For instance, 1:4 co-culture of HUVECs and fibroblasts results in large (650 μm in diameter) vascularized spheroids [37], while 5:1 co-culture of HUVECs and gingiva-derived progenitor cells (GPCs) enables a spheroid to sprout in hydrogel [38]. The first approach would be preferable to form large grafts, and the second one is preferable for bioprinting applications. The cells ratio must also be considered when creating a bone tissue. Culturing HUVEC and osteoblasts in the ratio of 5:1 has a positive effect on the angiogenesis, while the ratio of 1:5 promotes mineralization. To maintain the proliferation of aortic endothelial cells and MSCs in a co-culture, the balance should be shifted towards the ECs (5:1, respectively). Otherwise, osteogenic cells will suppress the proliferation of endothelial cells [36]. The same effect was shown for spheroids from adipose-derived stem cells (ADSCs) and HUVEC: the angiogenic potential was maintained at a ratio of 1:4, respectively [39]. In another study, a multiculture microfluidic platform was developed using HUVECs that were seeded in fibrin gels and cultured with human lung fibroblasts (HLFs). The engineered vessels containing perfusable lumens were formed inside the device [40].

HUVECs were also shown to be a good source of ECs in organoid vascularization. Since HUVECs are harvested from the fetal umbilical cord, their use is more compatible with the fetal or embryonic phase tissues that PSC-derived organoids resemble. Takebe et al. established the vascularized liver buds by co-culturing iPSC-hepatic endoderm cells, HUVECs, and human bone marrow mesenchymal stem cells (BM-MSCs). After the in vivo transplantation, further maturation was observed, and functional anastomosis developed from the host vessels into the liver buds within 48 h [41]. A recent study has developed an engineered tumor organoid model composed of HUVECs and mammary tumor cells. This approach allowed real-time and quantitative assessment of the tumor–vessel interactions with some conditions that mimic many in vivo properties. The imaging demonstrated that tumor organoids integrated into the endothelial cell lining, resulting in mosaic vessels [42].

Compared to HUVECs, endothelial colony-forming ECs (ECFC-ECs) have a superior vasculogenic potential to generate perfused networks [33]. Some studies showed that microvessels created using endothelial progenitor cells (EPCs) from adult blood are less stable than vessel networks formed with ECFC-ECs [34]. ECFC-ECs show a high angiogenesis capacity for paracrine interaction and regulation of the differentiation of other cells in a spheroid [32]. Co-culture of ECFC-ECs with ADSC reversed cellular senescence and demonstrated an improved therapeutic potential in wound healing [43]. EPCs have the same effect; they can stimulate osteogenic differentiation when cultured with bone marrow-derived MSCs [44].

There is also an option to use organ-specific endothelial cells, e.g., liver sinusoidal endothelial cells (LSECs), to achieve more specialized microvascular networks [45]. For example, using LSECs in organoid vascularization, Yap et al. generated hepatobiliary organoids with a liver-specific vasculature using mouse liver progenitor cells (LPCs) in combination with mouse LSECs. The LPC/LSEC organoids showed upregulation in the hepatic and biliary gene expression, more pronounced albumin secretion, and higher viability compared to LPC organoids over 7 days. This study revealed a novel method to generate vascularized hepatobiliary organoids, and both in vitro and in vivo results confirmed that the incorporation of LSECs with LPCs into organoids significantly increased differentiation of the hepatobiliary tissue in organoids and their survival post-transplantation [46]. Co-cultivation with ECs primarily stimulates the process of angiogenesis in vitro, and new blood vessels form from the existing ones. To stimulate vessels formation de novo (like vasculogenesis), co-differentiation with mesodermal progenitor cells can be used to stimulate the mesoderm-derived angioblasts’ differentiation. This approach is closer to the embryonic vasculature formation and can provide an opportunity to study interactions between different cells during the developmental stages. For example, vascularized pancreatic progenitors were obtained when human hPSCs were differentiated into the endodermal and mesodermal lineages, followed by their differentiation into pancreatic progenitors and ECs using VEGF. The pancreatic progenitors then efficiently differentiated into insulin-producing β-cells, as evidenced by the increased expression of β-cell markers and insulin secretion [47]. Another article described a detailed protocol used to obtain vascularized neural organoids by co-culturing with a mesodermal progenitor. iPSCs were differentiated in the neural and mesodermal direction in the 3D conditions, and then assembled organoids expressed neural markers after the maturation [48]. However, compared to using ECs to recapitulate vascularization, this method is more time-consuming, and the final percent of ECs is not so clear [24].

Choosing an approach to the internal induction should be based on the researcher’s goal. If the aim of experiments is to obtain vascularized constructs, then using a culture of HUVECs or EPCs is enough in most cases. However, in some cases, there is a need to use organ-specific ECs for the detailed analysis of cell cooperation in a specific tissue.

### 3.2. External Induction

**Figure 3:**
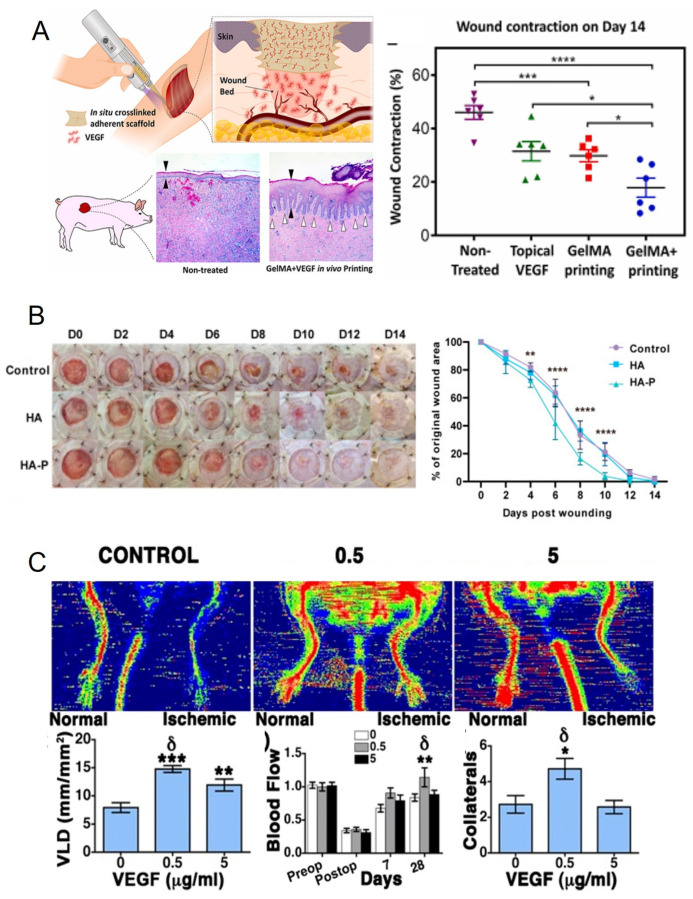
Application of external vascularization. (**A**) Wound healing by GelMA hydrogel supplemented with VEGF applied with printing and photo-crosslinking in situ. Using hydrogel provides a controllable release of VEGF and a prolonged effect on wound healing. General design of experiment (**left picture**) and wound contraction over time in control group, topical addition of VEGF, only GelMA and GelMA+VEGF (**right picture**). * *p* < 0.05, *** *p* < 0.0005, **** *p* < 0.00005. The images were adapted and changed from Ref. [50] according to http://creativecommons.org/licenses/by/4.0/ (assessed on 27 April 2025). (**B**) Utilization of recombinant peptides prominin-1-binding peptide (PR1P) to promote neovascularization and wound healing. PR1P can specifically bind to the VEGF receptor and promote capillary formation. Photo-crosslinked methacrylate hyaluronic acid (MeHA) bonded with PR1P (HA-P) significantly stimulates wound healing by promoting angiogenesis. Representative images of the healing process up to 14 days post-wounding (**left picture**) and wound closure curves of different groups (**right picture**). ** *p* < 0.01, **** *p* < 0.0001. The images were adapted and changed from Ref. [51] according to http://creativecommons.org/licenses/by/4.0/ (assessed on 27 April 2025). (**C**) Functional improvement of hind limb ischemia by α2-PI1–8-VEGF164. Fibrin hydrogels used for the delivery were stabilized by aprotinin to provide controllable and prolonged release of VEGF (0.5 and 5 μg/mL). Representative laser Doppler images of normal and ischemic limbs 28 d after treatment with control gels (control) or 0.5 μg/mL and 5 μg/mL α2-PI1–8-VEGF164 (first row). For vessel length density (second row, **left**), blood flow was measured by laser Doppler imaging (second row, **central**), and the number of collateral arteries was determined histologically in the adductor muscles (second row, **right**). *** *p* < 0.001 and ** *p* < 0.01 vs. negative control, * *p* < 0.05 vs. the 0.5 μg/mL condition. The images were adapted and changed from Ref. [52], according to http://creativecommons.org/licenses/by/4.0/ (assessed on 27 April 2025).

The external induction includes such methods as angiogenic induction with special media and growth factors, various hydrogels, or in vivo transplantation for pre-vascularization. Usually, this method accompanies the internal induction, but sometimes is applied independently. Often, both methods are used in combination; for example, a hydrogel can be used as a platform for pro-angiogenic growth factor delivery [49], or spheroids, encapsulated in a hydrogel, can become a transplant in the host for the in vivo vascularization. Different methods for external transplantation are presented below and summarized in Table 1. Some approaches used to stimulate external angiogenesis are described in Figure 3:

#### 3.2.1. Medium Composition

The first step in the angiogenic differentiation is the application of growth factors. VEGF, mainly VEGF-A, is the most common pro-angiogenic factor, which stimulates the migration and proliferation of ECs by anchoring VEGFR2 receptors. VEGF can also stimulate spheroids from adipose-derived stem cells (ADSCs) to grow sprouts in a hydrogel [53] and stimulate the angiogenic properties of those co-cultured with HUVECs spheroids. Fibroblast growth factors (FGF), mainly FGF-2, have an indirect effect on the angiogenesis, and can stimulate the proliferation of endothelial cells by the regulation of VEGFR1 alternative splicing in ECs [54] and influence the PDGF expression [55]. Angiopoietins 1 and 2 stimulate the maturation of new vessels by the stabilization of pericytes and smooth muscle cells. Interestingly, VEGF can convert the effect of Ang2 stimulation from an anti- to pro-angiogenic one. If VEGF is inhibited, Ang2 has the opposite effect and promotes endothelial cell death and vessel regression [56,57] Platelet-derived growth factor (PDGF) [58] and hypoxia-induced factor (HIFs) [59] also play a role in the vascular development and sprouting angiogenesis. It was shown that PDGF-C and its receptor PDGFR-a stimulate neovascularization. Overexpression of PDGF-C significantly improved the blood flow and vascularization in a model of ischemic disease [60]. HIFs have an indirect effect and stimulate transcription of response genes such as VEGF and Ang1 [61]. Several studies have shown that fetal bovine serum (FBS) can inhibit sprouting, while the vascularization potential is preserved [37,62]. Such culturing media are appealing not only from the angiogenic point of view, but also in terms of the clinical usage of tissue-engineered constructs.

#### 3.2.2. Hydrogel

Hydrogels in bioprinting not only provide a supportive scaffold for cell growth but also create the biomimetic microenvironment. By closely mimicking the ECM, hydrogels can facilitate better cell adhesion, proliferation, migration, and differentiation [63]. Additionally, the properties of hydrogels such as their stiffness, porosity, and degradation rates enable one to customize the bioprinting process to meet the specific tissue engineering requirements and influence formation of vessels. Although hydrogels are mainly used in combination with growth factors to stimulate vascularization [49], there are some examples confirming that the hydrogel itself or a fragment of its degradation can stimulate vascular formation [64]. For example, during degradation, hyaluronic acid (HA) produces small hyaluronan oligosaccharides molecules which can stimulate angiogenesis [65,66]. It was shown that HA can facilitate the angiogenesis of ECFC combined with MSCs [66]. Moreover, the HA effect on endothelial cells depends on the molecular weight, and only high-molecular-weight (HMW) HA can increase the tube formation in cultured human cerebral microvascular ECs (HCMVECs) [67]. Other polysaccharide-based hydrogels (alginate, chitosan) can also be used as proangiogenic stimuli, but often combined with growth factors [49].

Of protein-based hydrogels, human platelet lysate (HPL) has the best effect on angiogenesis [68]. Culture of ECFCs in HPL stimulate the formation of sprouts and cell proliferation, since it contains VEGF-A, PDGF-BB, and FGF-2 [68]. Other protein-based hydrogels, such as fibrin and collagen, can also stimulate angiogenesis, but this effect is not so pronounced [69]. Moreover, the mechanical characteristics and plasticity of the hydrogel play an important role in stimulating angiogenesis, since they affect the ability of cells to remodel the matrix and migrate into the thickness of the hydrogel. Fibrin hydrogel is known to promote angiogenesis by triggering and supporting the formation of endothelial networks [70,71], and the modification of a fibrin gel with polyethylene glycol (PEG) can increase the proangiogenic effect [64]. Different combinations of hydrogels, such as collagen-chitosan [72] or collagen-HA [70], showed great results in angiogenesis promotion.

While growth factors and hydrogels can individually stimulate vascularization, their combination yields the best results. For example, GelMA hydrogel supplemented with VEGF can stimulate wound healing and vascularization, and provides better results compared to the topical use of VEGF itself (Figure 3A) [50]. The high efficacy of hydrogel as a delivery platform can be explained be its ability to prolong the release of growth factors. For example, the addition of aprotinin to fibrin gel can stabilize it and prolong the release of VEGF [52]. This approach demonstrates excellent results for the treatment of hind limb ischemia [52] (Figure 3C). Also, instead of a short half-life and unstable VEGF, recombinant peptides can be used to promote neovascularization and wound healing. For example, prominin-1-binding peptide (PR1P) can specifically bind to the VEGF receptor and promote capillary formation. Photo-crosslinked methacrylate hyaluronic acid (MeHA) bonded with PR1P can significantly stimulate wound healing by promoting angiogenesis [51] (Figure 3B).

In some studies, in vivo transplantation is also considered a method for external induction. However, the ability of organoids and spheroids to integrate into tissues after the transplantation depends on their cellular composition and the use of external stimulation, such as growth factors or specific hydrogels, which were previously described. Transplantation can only confirm the vasculogenic potential of a bioequivalent, but cannot be used as a method to stimulate it.

## 4. Applications of Vascularized Spheroids and Organoids Using 3D Bioprinting

Naturally, prevascularized spheroids are appealing to use in 3D bioprinting. Spheroids, precisely positioned in the supporting hydrogel, can produce prevascularized microtissues, which develop to branched vascular trees in macrotissues in vivo. Prevascularized co-culture spheroids including ADSCs and HUVECs are perfect candidates for the use as building blocks in microvasculature bioprinting [73]. These objects can survive in large-scale tissue-like constructs while organizing the microvasculature locally at the micro-levels, and are often used for 3D extrusion bioprinting. For instance, De Moor et al. developed 3D-bioprinted highly viable vascular patches, anastomosed with the chicken embryo’s vascular network [5]. Such constructs can be applied to restore the volume and function of soft tissues, e.g., adipose tissue. Self-differentiation in the adipogenic and angiogenic direction alleviates the graft integration and tissue regeneration [73].

Nowadays, bioprinting with vascularized spheroids is applicable for different types of organs and tissues such as the liver [41,45,46,74,75,76], intestine [77], kidney [78,79], brain [41,80,81], heart [82], bone [83], tumors, and other tissues.

This review focuses more on 3D bioprinting and the modeling of vascularized healthy tissues. However, angiogenesis is also important for the modeling of certain pathological conditions. For example, reproducing the specific vascular environment is crucial for modeling the tumor microenvironment. Several recently published papers show that combining 3D bioprinting and spheroid culture seems appealing for the efficient drug testing and investigation of the tumor invasion and vascularization mechanisms [84,85]. Promising results were obtained in the field of breast cancer modeling [86,87]. However, tumor modeling and vascularization is a complex problem which requires a separate review to discuss.

A more detailed explanation of the use of bioprinting with spheroids and organoids to create vascularized healthy tissues is provided below and summarized in Table 2. In this review, we did not examine the bioprinting of all organs; rather, we focused on those that have been the subject of extensive research to gain a better understanding of the potential for the widespread use of vascularized spheroids and organoids.

### 4.1. Bone

The bone vasculature is formed through angiogenesis. The most important part of the bone responsible for the angiogenesis is the metaphysis, which is located at the ends of long bones. It contains a rich network of arteries that supply blood to the growing bone during development and play a critical role in the repair and remodeling processes. Other parts of the bone, such as the diaphysis and periosteum, receive blood supply from the central nutrient artery and periosteal arteries, respectively [96]. Thus, angiogenesis is one of the critical conditions for the successful development of engineered bone grafts and bone tissue regeneration.

In addition to the function of supplying bone tissue, endothelial cells also play an important role in paracrine stimulation, and secrete many factors that can trigger the process of osteogenic differentiation. Co-cultures of osteogenic and angiogenic cells can significantly stimulate both differentiation potentials. For osteogenic differentiation, MSCs from different sources are mainly used, and rarely osteoblasts. For angiogenic differentiation, HUVECs [97], ECPs [36], and mature ECs [39] are used. In these types of co-cultures, the ratio between cells and order of differentiation is especially important. It was shown in many studies that, for angiogenic potential, the predominance of angiogenic rather than osteogenic cells is crucial [36], and angiogenic induction should be performed first [98], since high mineralization after osteogenic induction can inhibit the formation of blood vessels. A recent study demonstrates that adipose tissue-derived microvascular fragments (MVF) can also be a great source of buildings blocks for bone tissue engineering when cultured with osteoblasts [88]. Using aspiration-assisted bioprinting (AAB) and spheroids from MSC and HUVECs, mature bone bioequivalents were produced, and the best maturation was obtained when osteogenic induction was performed before angiogenic induction [89]. In another study, to form a vascularized bone bioequivalent, osteogenic differentiation of MSCs was first performed, followed by the HUVECs spheroid seeding in the inner, soft GelMA-based bone marrow [90]. Since ADSCs have both osteogenic and angiogenic potential, it is a promising tool for bone tissue engineering [53]. In a recent study, ADSC was differentiated into endothelial and osteogenic progenitors, assembled into spheroids and precisely positioned with aspiration-assisted bioprinting (AAB) to form a Harversian canal model—small tubes in the cortical bone, where blood vessels and nerves are located [83]. Post-printing spheroid fusion resulted in the development of a heterotypic osteogenic/angiogenic structure. In another study, AAB were used for bioprinting of a bone tissue equivalent from MSCs and HUVEC. Cells were cultured at the ratio of 1:1, predifferentiated in the osteogenic direction for 10 days, and then used for bioprinting [89]. ADSCs incapsulated in porcine bone-derived dECM (BdECM), β-tricalcium phosphate (b-TCP), and ECs spheroids demonstrated outstanding angiogenesis and osteogenic activities after bioprinting and after the in vivo transplantation in the rat obliterated mastoid model [31]. Thus, using different co-cultured spheroids and organoids for bioprinting can be a promising tool for the formation of a large-size highly vascularized bone equivalent. There is a small selection of studies where co-culture has been used to create a vascularized bone bioequivalent, and a more in-depth review may be necessary to cover this topic in more detail.

### 4.2. Liver

The liver plays the central role in many physiological processes, and its diseases are among the major causes of mortality worldwide, leading to around 2 million deaths annually [99]. The liver has a highly vascularized tissue, and, for its proper functioning, integration into the recipient’s vascular network after transplantation is crucial. To achieve vascularization, HUVECs are mostly used, but in some cases, liver sinusoidal endothelial cells (LSECs) are better to achieve higher tissue specificity. Almost half of the liver models use HepG2 cells, and the second most frequently used cells are primary hepatocytes [100]. But in some cases, hepatic progenitor cells (HPC) [101], or even MSCs, are used for the liver construction [102].

In a recent article [91], a combined approach was used to achieve vascularization within a bioequivalent. Self-organized spheroids from primary mouse hepatocytes and endothelial cell line C166 were printed on the surface of a pre-formed polymer network, which contained vascular analogs that were preliminarily populated with spheroids from endothelial cells. The formed liver bioequivalent was fully vascularized, expressed characteristic markers, and stimulated neovascularization after the transplantation in mice [91]. Another successful example of obtaining vascularized liver constructs using bioprinting was represented by the study, in which hepatic lobule arrays on a large scale (~1 mm) were printed using the extrusion bioprinting technique [75]. The constructed hepatic lobules contained hepatic cells, endothelial cells, and a lumen, they were highly vascularized and showed increased albumin secretion, urea production, and albumin, MRP2, and CD31 protein levels, as well as cytochrome P450 enzyme activity compared to the mixture of hepatic and endothelial cells [75]. A combination of mouse liver progenitor cells (LPCs) and mouse liver sinusoidal endothelial cells (LSECs) led to the formation of polygonal hepatocyte-like cells and biliary ducts over a 7-day period and the obtained liver organoids had upregulation of liver-specific genes such as albumin, CPS1, CYP3A11 [46]. Liver organoids from human primary hepatocytes, namely ESC and iPSC, demonstrate high expression of the endothelial markers CD54 and CD31, stimulate neovascularization, and produce albumin after transplantation under rodent kidney capsule (Figure 2B,C) [30]. Although most of the liver models use HepG2 or primary hepatocytes [100], there are several reports of MSCs being used as an alternative source. For example, small-size (50 µm) hADSC spheroids show great pro-angiogenic effects, and HUVECs/hADSCs co-culture in the ratio of 2:1 promotes hepatic differentiation and vascularization in vitro and in vivo, especially when HUVECs are patterned into hexagons [102]. Thus, different spheroids can be an effective tool for the formation of a liver tissue bioequivalent.

### 4.3. Cardiac Tissue

Cardiac tissues require a constant supply of oxygen and nutrients to function effectively, and the lack of a vascular supply can cause hypoxia-induced cardiomyocyte death resulting in fibrosis and tissue degeneration. The latter are the main causes of cardiovascular diseases (CVDs), which continue to be the leading cause of death worldwide [103]. Tissue engineering has shown promising results in the regeneration of heart tissue, but for better integration into the host tissues, high vascularization is essential. Generally, to restore the heart function, cardiomyocytes (CM) or cardiac fibroblasts (FB) with ECs are used. Since CM have a limited proliferative potential, MSCs, ESCs, and iPSCs are potential sources for both experimental and clinical regenerative applications. Co-cultures of HUVECs or human coronary ECs [94] with cardiomyocytes obtained from iPSCs or ESCs or with cardiac FB are the most common combination for heart engineering. Co-culture of iPSC-CMs with FB, ADSCs, and HUVECs resulted in the formation of lumen-like structures and a Z-line. Spontaneous beating and verapamil and isoproterenol responsiveness were observed [93]. Similar results were obtained when spheroids from cardiomyocytes, HUVECs, and FBs were cultured on microfluidic chips (µFCs) with perivascular cells, known as pericytes. Cardiac spheroids were perfusable, and formed lumen and synthesis cTnT (cardiac troponin T), NG2 (pericyte marker), and CD31 (ECs marker) [29]. Sacrificial bioprinting was used to obtain a functional cardiac tissue from iPSC-derived CMs and FBs with HUVECs. Gelatin was used as a sacrificial ink to form the vessels’ chambers. The created heart showed contractile activity and a sacromeric architecture [93]. Cardiac spheroids from FB, ECs, and myocytes in the ratio of 1:1:2 after bioprinting and VEGF stimulation also demonstrated spontaneous contraction [94]. In a recent study, Liu et al. showed the possibility to restore the cardiac function via the engraftment of spheroid-based 3D bioprinted grafts. Microvasculature transplant seeds were formed from early vascular cells (EVCs)-derived hESCs and bioprinted in a fibrin-based hydrogel on the surface of a sacrificial gelatin gel. The formed patches were transplanted to ischemic regions of myocardial infarction (MI) in mice models and resulted in the reduced fibrosis area and ameliorated cardiac function [95]. The bioprinted heart tissue can also function as an in vitro platform, offering insights into human physiological responses. Freeform reversible embedding of suspended hydrogels (FRESH) in bioprinting was used to organize hiPSC-CMs, ECs, and FB in the functional cardiac tissue. The created construct responded to isoproterenol (β-adrenergic receptor agonist) and verapamil (calcium channel blocker). The same response was observed when iPSC-CMs were co-cultured with FB, ADSCs, and HUVECs [92]. Thus, the bioprinting technology using spheroids and organoids represents a promising approach for the development of clinically relevant cardiac constructs and the management of myocardial infarction and its associated complications. It also provides a promising model for drug testing.

## 5. Challenges and Future Prospects

The integration of a functional vascular system into tissue-engineered constructs remains one of the most significant challenges in the field of biofabrication [104]. The major issue is our incomplete understanding of the molecular pathways that govern the vasculature development and self-organization, especially in the organism. While various growth factors and signaling molecules have been identified, the intricate interplay between these components and their role in promoting angiogenesis, ECM remodeling, and immune reactions remains poorly understood. Additionally, the complexity of cellular interactions within the tissue microenvironment poses another challenge. Achieving the right balance of cell types, including endothelial cells, pericytes, and supporting stromal cells, is essential for creating stable and functional vascular networks. The heterogeneity of cell populations within organoids can lead to unpredictable outcomes, complicating efforts to maintain viability and functionality over time. Moreover, the protocols required for bioprinting and assembling these structures are often complex and require precise control over multiple variables, such as cell density, growth factor gradients, and scaffold properties [21]. Furthermore, while current vascularization strategies can generate capillary-like structures in vitro, the transition to integrating these networks with the host’s vascular system remains a critical barrier. Successful anastomosis—where the engineered vessels connect seamlessly with existing blood vessels—is crucial for ensuring the long-term survival and functionality of bioengineered tissues. Moreover, the key for successful regeneration is interaction between the regenerating tissue, blood vessels, and nerves. Without effective integration, even the most sophisticated constructs may fail to survive once implanted.

Despite these challenges, several promising directions could significantly enhance our ability to create vascularized tissue-engineered constructs. The concept of building blocks is highly promising for this goal, since it provides the ability to ensure the self-organization of capillary-like structures at the micro level. With the support of the proper environment (hydrogel) and channeling technologies (3D bioprinting), spheroids and organoids can fuse into the vascular networks throughout the whole construct and support the high viability of the whole structure. Also, organoids represent modular pre-matured tissue equivalents that are more stable and viable, while having a necessary number of various cell types. By engrafting these organoids in vivo, they can survive, grow, and connect to host vasculature [105]. Recent studies show that preconditioning of the future organ helps it to develop required mechanical properties, cell differentiation state, and general functionality [93,106,107]. Such bioreactors can provide controlled environments that mimic physiological conditions, allowing for the perfusion of nutrients and oxygen while simultaneously applying mechanical stimuli [108]. This approach not only supports cell viability, but also encourages the maturation of vascular networks, improving their functionality and integration potential [109]. Additionally, the implementation of bioreactors equipped with microfluidic systems presents an exciting opportunity to direct vascular development, particularly for the creation of vascularized organs. For example, Campisi et al. have made the blood–brain barrier on a chip consisting of iPSCSs-derived endothelial cells, pericytes, and astrocytes [110]. Resulted microvasculature have shown the formation of vessel-like structures, tight junctions, and deposited ECM while slightly inhibiting the permeability of the microfluidic channels. Another prospect involves the use of mathematical modeling to predict and optimize the self-organization processes of vasculature. For example, such models exist for the prediction of spheroids fusion, mechanical properties, the processes of cell migration in spheroids, and oxygen distribution [111,112,113]. Several studies have shown the ability to model blood flow, including the diameter of the vessel and personal differences [114,115]. Increasingly, AI/ML models are becoming more commonly used to extract new biological data and use them in research [116]. By simulating the dynamics of cell interactions and vascular network formation, researchers can gain insights into how to manipulate the conditions necessary to promote more effective vascularization. Additionally, the implementation of bioreactors equipped with microfluidic systems presents an exciting opportunity to direct vascular development. Moreover, advancements in software tools for monitoring and controlling the bioreactor conditions can further enhance our ability to engineer vascularized tissues. The real-time data analysis can help researchers to fine-tune parameters such as flow rates, shear stress, and nutrient concentrations, enabling them to optimize the growth and organization of vascular structures within the constructs.

Thus, while significant challenges remain in achieving effective vascularization in tissue engineering, innovative approaches, such as mathematical modeling, bioreactor technology, and advanced monitoring systems hold great promise for overcoming these obstacles. By harnessing these tools, researchers can pave the way for more reliable and functional bioengineered tissues that can successfully integrate into host systems for long-term therapeutic applications.

## 6. Conclusions

The use of spheroids and organoids as building blocks in the engineering of vascularized tissues represents an emerging technology in the field of 3D bioprinting, since they can resemble the 3D structure, specific cellular functions, and cell–cell and cell–ECM interactions most comparable to the real organ. Biofabrication technologies, namely 3D bioprinting, seem a promising tool for the organization of these building blocks in a controllable manner to shape future channels and networks. This approach allows for the combination of a wide variety of components, including cells, growth factors, and hydrogels, to promote the bioequivalent’s vascularization. Generally, two main approaches are used to stimulate vascularization: internal and external induction. Internal induction focuses on selecting the optimal cell types for co-differentiation. Co-culturing with endothelial cells or their precursors is the most commonly used method to stimulate vascularization, also playing a crucial role in paracrine regulation, which can trigger cell differentiation. External induction involves the use of specific growth factors, media, and various types of hydrogels. These methods are often employed in conjunction to achieve the optimal vascularization results. Typically, the external induction complements the internal induction; for example, hydrogels can serve as platforms for the delivery of pro-angiogenic growth factors, while spheroids can be encapsulated within these hydrogels. With the ongoing progresses in the integration of the vascular system into 3D-bioprinted organoids, the bioprinting technique is considered as one of the most promising methods to create a vascularized tissue for in vitro applications, such as drug screening and high-throughput assays, as well as implantation for the replacement or support of the damaged tissue.

## Figures and Tables

**Table 1:** Methods of external induction.

Medium compounds	VEGFs (VEGF-A)	Direct effect - Stimulate the migration and proliferation of the ECs
	FGFs (FGF-2)	Indirect effect - Stimulate the proliferation of the ECs by regulation of VEGFR1 alternative splicing - Stimulate PDGF expression
	PDGFs (PDGF-C)	Direct effect - Stimulate neovascularization
	HIFs	Indirect effect - Stimulate transcription of VEGF and Ang1
	Ang1	Indirect effect - Stimulate maturation of the new vessels by stabilizing pericytes and SMCs
Hydrogel	Hyaluronic acid	- The mechanism of action is unknown
	Human platelet lysate	Direct effect - Contains VEGF-A, PDGF-BB and FGF-2
	Fibrin hydrogel	- The mechanism of action is unknown

**Table 2:** Application cases for vascularizing via organoids and spheroid.

Induction Method	Approach	Cell Types	Biomaterial	Results	Tissue	Ref.
Internal	Co-culture with ECs	ADSCs and HUVECs	BdECM b-TCP	ADSCs in BdECM and b-TCP + ECs spheroids ↑ angiogenic and osteogenic activities ↑ vascularization in rat obliterated mastoid model	Bone	[31]
External	miR transfection	ADSCs	**-**	ADSC differentiated into endothelial and osteogenic progenitors + AAB	Haversian bone canal	[83]
Internal	Co-culture with ECs	Adipose tissue-derived microvascular fragments Osteoblasts	Collagen	↑ sprouts formation ↑ vascularization after in vivo transplantation in the dorsal skinfold chamber model	Bone	[88]
Internal	Co-culture with ECs	MSC and HUVECs	**-**	↑ osteogenic differentiation and bioequivalent maturation + AAB → osteogenic induction → angiogenic induction	Bone	[89]
Internal	Co-culture with ECs	MSC and HUVECs	GelMA	MSCs → osteogenic differentiation + HUVECs spheroid seeding in the inner soft GelMA-based bone marrow	Bone	[90]
Internal/External	Co-culture with ECs	iPSC-HEs HUVECs MSCs	Matrigel	↑ expression of liver markers ↑ albumin after transplantation	Liver	[41]
Internal	Co-culture with ECs	Mouse LPCs and LSECs	-	LPCs and LSECs co-culture after 7-day ↑ upregulation of liver-specific genes (albumin, CPS1, CYP3A11)	Liver	[46]
External/ Internal	Co-culture with ECs, sacrificial materials	HepG2/C3A and ECs	Alginate Collagen Gelatin	↑ vascularization ↑ albumin secretion ↑ urea production ↑ cytochrome P450 enzyme activity	Liver	[75]
Internal	Co-culture with ECs	Primary mouse hepatocytes ECs line C166	-	Hepatocytes + ECs spheroids printed on the surface of a preformed polymer network, which were preliminarily populated with ECs spheroids ↑ vascularization ↑ expression of characteristic markers ↑ neovascularization in mice	Liver	[91]
Internal	Co-culture with ECs	MSCs, iPSC-HEs (iPSC-derived hepatic endoderm cells) and HUVECs	Matrigel	↑ integration in host vasculature Secretion of liver proteins after transplantation under cranial window	Liver	[76]
External/ Internal	Co culture with ECs	HUVECs, Neonatal rat cardiomyocytes or hiPSC-CM	Alginate GelMA	↑ sarcomeric α-actinin (protein responsible for contractile function) ↑ connexin-43 (inter-cellular conductive function) Synchronous beating	Cardiac tissue	[82]
Internal	Co-culture with ECs	iPSC-CMs, FB, ADSCs, HUVECs		Lumen-like structures, Z-line Spontaneous beating Verapamil and isoproterenol responsiveness	Cardiac tissue	[92]
Internal	Co-culture with ECs	iPSC-CMs, FB, HUVECs	Gelatin (sacrificial bioink)	Sacromeric architecture Contractile activity	Cardiac tissue	[93]
External/ Internal	Co-culture with ECs VEGF	FB, ECs, and myocytes	-	Spontaneous contraction	Cardiac tissue	[94]
External/Internal	Co-culture with ECs VEGF FGF-2	hESCs-EVCs CMs	Collagen I Matrigel	Spontaneous beating Organized vascular network Cardiac function recovery after MI in mice	Cardiac tissue	[95]

## Data Availability

No new data were created.

## Abbreviations

ADSCs	Adipose-derived stem cells
MSCs	Mesenchymal stem cells
BM-MSCs	Bone marrow mesenchymal stem cells
BdECM	Bone-derived extracellular matrix
b-TCP	Β-tricalcium phosphate
CVDs	Cardiovascular diseases
ECM	Extracellular matrix
DPSCs	Dental pulp stem cells
EVCs	Early vascular cells
ECs	Endothelial cells
ECFC	Endothelial colony–forming cell
EPCs	Endothelial progenitor cells
ESC	Embryonic stem cells
FBS	Fetal bovine serum
FGF-2	Fibroblast growth factors
FBs	Fibroblasts
FRESH	Freeform reversible embedding of suspended hydrogels
GelMA	Gelatin methacryloyl
GPCs	Gingiva-derived progenitor cells
HMW	High molecular weight
hPSCs	Human pluripotent stem cells
HLFs	Human lung fibroblasts
HPL	Human platelet lysate
hTMSCs	Human turbinate mesenchymal stem cells
HUVECs	Human umbilical vein endothelial cells
HA	Hyaluronic acid
HIFs	Hypoxia induced factor
iPSC	Induced pluripotent stem cells
LPCs	Liver progenitor cells
LSECs	Liver sinusoidal endothelial cells
MVF	Microvascular fragments
MI	Myocardial infarction
PDGFR	Platelet-derived growth factor receptor
PDGF	Platelet-derived growth factor
PLGA	Poly (lactic-co-glycolic acid) copolymer
PEG	Polyethylene glycol
SMCs	Smooth muscle cells
SLA	Stereolithography
TGF-b	Transforming growth factor beta
UC-MSC	Umbilical cord mesenchymal stem cells
VEGF	Vascular endothelial growth factor
CM	Cardiomyocytes
